# Engineering Oilseed Microbiome Synergy for Saline Alkaline Soil Restoration

**DOI:** 10.3390/plants14142197

**Published:** 2025-07-16

**Authors:** Shijie Ma, Tong Tang, Chang Du, Zheng Yang, Binjie Gan

**Affiliations:** 1 Crop Research Institute, Anhui Academy of Agricultural Sciences, Hefei 230031, China; mashijieaaas@163.com; 2 Department of Computer Science and Information Technologies, Elviña Campus, University of A Coruña, 15001 A Coruña, Spain; tongtang@scu.edu.cn; 3 Guangdong Provincial Key Laboratory of Biotechnology for Plant Development, School of Life Science, South China Normal University, Guangzhou 510631, China; duchang@m.scnu.edu.cn; 4 Zhengzhou Research Base, State Key Laboratory of Cotton Bio-Breeding and Integrated Utilization, School of Agricultural Sciences, Zhengzhou University, Zhengzhou 450001, China

**Keywords:** artificial intelligence, microbiome, oil crop, soil restoration

## Abstract

Soil salinization poses a critical threat to global agriculture, necessitating innovative strategies for sustainable remediation. This review synthesizes advances in leveraging plant–microbe interactions to remediate saline–alkali soils, focusing on oilseed crops—*Brassica napus*, *Glycine max*, *Arachis hypogaea*, *Helianthus annuus*, and *Sesamum indicum*—as keystone species for ecosystem restoration. These crops exhibit unique adaptive strategies, including root architectural plasticity and exudate-mediated recruitment of stress-resilient microbiomes (Proteobacteria, Actinobacteria, and Ascomycota), which collectively stabilize soil structure and enhance nutrient cycling, ion homeostasis, and soil aggregation to mitigate soil salinity and alkalinity. Emerging technologies further amplify these natural synergies: nanomaterials optimize nutrient delivery and microbial colonization, while artificial intelligence (AI) models predict optimal plant growth-promoting rhizobacteria (PGPR) combinations and simulate remediation outcomes. This integration establishes a roadmap for precision microbiome engineering, offering scalable strategies to restore soil health and ensure food security in saline–alkali ecosystems.

## 1. Introduction

Soil salinization is a pressing global environmental issue that severely threatens agricultural production and food security [1,2]. Currently, approximately 20% of cultivated land and 33% of irrigated areas worldwide are affected by salinization, with climate change and unsustainable agricultural practices such as poor irrigation and deforestation likely to exacerbate this trend [3,4]. Salinization arises through natural processes such as climatic and hydrological dynamics or anthropogenic activities, characterized by the accumulation of soluble salts, including sodium chloride, calcium sulfate, and magnesium sulfate, which elevate soil electrical conductivity [5]. This process degrades soil structure through reduced permeability and aeration, induces soil dispersion and structural collapse, and may lead to metal toxicity and nutrient deficiencies [6,7,8,9,10]. These changes significantly impair cropland productivity, particularly in arid and semi-arid regions where high evapotranspiration rates accelerate salt accumulation. Subsequently, salt stress directly inhibits plant root water uptake and metabolic activity through osmotic imbalance and ionic toxicity, leading to reduced photosynthesis, stunted growth, and lower crop yields [6,11,12]. Globally, an estimated three hectares of arable land are lost to salinization every minute, potentially resulting in a 30% reduction in food-producing areas within the next 25 years [13,14].

Traditional approaches to saline soil management, including physical drainage and chemical amendments, often prove insufficient and environmentally unsustainable [15]. This has driven the development of innovative biotechnological solutions that harness the synergistic potential of plant–soil–microbe interactions for sustainable soil remediation [16]. These emerging strategies integrate multiple biological components, including specialized crop cultivation, engineered microbial communities, and advanced nanomaterial applications, representing a paradigm shift toward eco-friendly saline soil restoration [17].

Oilseed crops, particularly rapeseed (*Brassica napus*), have gained attention for their adaptability to saline–alkali soils and soil remediation potential [18]. These crops thrive in diverse saline–alkali environments, utilizing extensive root systems to absorb soil salts and reduce salt concentrations through transpiration. They significantly lower soil pH, total salt content, and sodium ion (Na^+^) levels while enhancing available phosphorus and potassium [19]. The plant–soil–microbe symbiotic relationship forms the cornerstone of this remediation approach, where tripartite interactions create synergistic effects that exceed the sum of individual components [20]. These interactions enhance nutrient cycling, improve soil structure, and mitigate salt stress through coordinated mechanisms involving root exudates, microbial activity, and soil physicochemical changes. Root architecture–microbe interactions constitute a key remediation mechanism: specialized root traits such as deep rooting improve nutrient acquisition, while rhizosphere microbiome modulation enhances stress resilience [21].

Recent advances in microbial community engineering have demonstrated that structured microbial consortia can be rationally designed to enhance salt tolerance and promote soil restoration functions. The application of high-throughput sequencing technologies has unraveled the complexity of rhizosphere microbiomes, enabling the identification of keystone microbial species that govern community assembly and ecosystem functioning under saline stress [22]. These insights pave the way for the targeted manipulation of microbial communities to improve plant resilience and rehabilitate saline soils sustainably [23]. For instance, inoculation with *Pseudomonas fluorescens* reshapes the rhizosphere microbial community of oilseed crops, promoting plant growth and nutrient cycling [24].

The integration of nanomaterials and AI into biological remediation systems represents a cutting-edge approach that amplifies the efficiency of plant–microbe interactions [17,25]. Engineered nanoparticles, including biochar nanocomposites and metal oxide nanoparticles, serve as carriers for beneficial microorganisms while simultaneously improving soil physical properties and nutrient retention [26]. These nanoscale interventions can precisely modulate rhizosphere chemistry, enhance microbial colonization, and provide a controlled release of bioactive compounds [17].

The synergy between oilseed crops and rhizosphere microbes alleviates salt–alkali stress through multifaceted pathways: root exudates such as organic acids recruit beneficial microbes including *Bacillus subtilis* to enhance nitrogen and phosphorus bioavailability, optimizing soil nutrient cycling [27,28]. Concurrently, plant growth-promoting rhizobacteria (PGPR) mitigate salt-induced oxidative damage by scavenging reactive oxygen species (ROS), maintaining plant metabolic homeostasis [28]. Furthermore, oilseed cultivation significantly increases microbial diversity in saline–alkali soils [29,30], where high-complexity communities enhance nutrient utilization and ecological resilience to counteract salinity impacts [31]. These coordinated mechanisms provide a systemic biological strategy for saline–alkali soil bioremediation.

## 2. Oilseed–Microbiome Synergy Under Saline–Alkali Stress

The adaptive capacity of oilseed crops to reconfigure their root systems under environmental stress is increasingly recognized. This plasticity extends beyond root elongation or branching, encompassing intricate interactions between root architecture and exudate composition that shape the rhizosphere microenvironment [32,33]. Transcriptomic analyses have revealed that salt stress triggers differential expression of genes controlling root development, including auxin response factors (*ARFs*) and lateral organ boundary domain (*LBD*) genes, which orchestrate morphological remodeling in response to saline conditions [34,35]. Research reveals multidimensional salt–alkali stress response mechanisms through root morphological remodeling, with exudate-mediated rhizosphere regulation being particularly critical. Enhanced vertical root elongation under salt stress serves as a key adaptive mechanism affecting plant functionality and stress resistance in oilseed crops. This root adaptation proves vital in saline–alkali environments by expanding the effective zone for water/nutrient acquisition and mitigating the adverse effects of surface salt accumulation [36,37].

Excessive fertilization with compounds such as nitrate and ammonium or irrigation with sodium-rich water leads to alkaline salt accumulation, including sodium carbonate and sodium bicarbonate, which elevates soil pH through hydroxyl ion release during hydrolysis [5,38]. Root systems counteract this through organic acid secretion, such as malic and citric acids, which chelate cations, regulate rhizosphere pH, and enhance nutrient uptake efficiency. Concurrently, root-derived phenolic compounds alleviate oxidative damage via ROS scavenging while modulating soil pH to recruit beneficial microorganisms, thereby improving soil health [39,40,41].

Oilseed crops demonstrate varying salt–alkali tolerance levels [42,43]. Transcriptomic profiling of *Brassica napus* under salt stress reveals an upregulation of sodium/hydrogen antiporter (*NHX*) genes and high-affinity potassium transporter (*HKT*) family members, enabling Na^+^ compartmentalization and K^+^/Na^+^ homeostasis, which contribute to salt tolerance and soil amelioration [44]. In *Glycine max*, RNA-seq analysis indicates that the MYB68 transcription factor enhances salt stress resistance by regulating genes involved in osmoregulation, potentially including aquaporin genes (*PIPs* and *TIPs*), and supports photosynthetic efficiency through pathways that may involve ribulose-1,5-bisphosphate carboxylase/oxygenase (RuBisCO) activase [45]. Integrated metabolomic and transcriptomic analyses in *Helianthus annuus* demonstrate proline accumulation through upregulated pyrroline-5-carboxylate synthetase (*P5CS*) expression and the maintenance of K^+^/Na^+^ homeostasis via differential regulation of Shaker-type potassium channels under salt stress [46]. In *Sesamum indicum*, transcriptomic analyses suggest molecular coordination through co-expression networks linking plasma membrane H^+^-ATPases with antioxidant systems, including superoxide dismutase (*SOD*) and catalase (*CAT*) gene families, to enhance salt stress tolerance [47]. Comparative transcriptomics in *Arachis hypogaea* under salt stress reveals optimized photosynthate allocation through the differential expression of sucrose transporter genes and potential modulation of root architecture via gravitropic response genes [48].

The coordinated root exudate–morphology responses suggest plant–microbiome synergy as a crucial stress mitigation strategy. Rhizosphere microorganisms participate directly in pH regulation and salt detoxification through exudate metabolism, ion homeostasis modulation, and bioactive compound synthesis. Their structural and functional dynamics may play pivotal roles in saline–alkali soil remediation.

## 3. Rhizosphere Microbiota Drive Soil Remediation

The relationship between rhizospheric microbial communities and oilseed crops is critical for enhancing plant growth and resilience, particularly in saline–alkali environments. Functional trait-based approaches prioritize microorganisms based on specific capabilities rather than taxonomic identity, enabling targeted remediation strategies.

In *Brassica napus*, root-associated bacterial communities are dominated by the phyla Proteobacteria, Actinobacteria, Acidobacteria, and Gemmatimonadetes, while fungal communities primarily comprise the phyla Ascomycota, Basidiomycota, Chytridiomycota, and Zygomycota [49]. In *Glycine max*, bacterial communities are enriched with Bradyrhizobium and Pseudomonas (both Proteobacteria) and Bacillus (Firmicutes), with fungal communities dominated by Ascomycota and Basidiomycota [50]. Similar bacterial profiles (Bradyrhizobium, Pseudomonas, and Bacillus) are observed in *Arachis hypogaea* and *Glycine max* [51]. *Helianthus annuus* roots predominantly host Acidobacteria and Saccharibacteria, whereas stems are colonized by Proteobacteria, Bacteroidetes, and Gemmatimonadetes [52] (Table 1).

Bacterial communities in oilseed crops play pivotal roles in saline–alkali soil remediation. Under salt–alkali stress, core rhizobacteria such as Proteobacteria secrete organic acids including citrate and oxalate to solubilize immobilized phosphate, alleviating phosphorus fixation [53,54,55]. Specific strains such as Rhizobium enhance nitrogen availability through symbiotic nitrogen fixation with legumes [56,57]. Actinobacteria degrade complex organic compounds such as cellulose and chitin and chelate excess metal ions, mitigating ionic toxicity [58,59,60], while antibiotic-producing strains such as Streptomyces suppress soil-borne pathogens to bolster plant resilience [61,62]. Salt-tolerant Acidobacteria maintain metabolic activity via osmoregulation and promote soil aggregate formation to improve structure [63,64]. Gemmatimonadetes accelerate organic matter mineralization, reducing soil alkalinity and enhancing micronutrient availability [65,66].

Fungal communities in oilseed crops contribute synergistically to soil amelioration. Ascomycota secretes biomass-degrading enzymes to release humic substances, improving water retention [67,68]. Trichoderma enhances plant antioxidant defenses [69,70], while Penicillium improves salt tolerance by facilitating nutrient uptake [71,72]. Basidiomycota, including arbuscular mycorrhizal fungi (Glomus), expand root nutrient acquisition and stabilize soil aggregates via glomalin-related soil protein (GRSP) secretion, counteracting salt-induced hardening [73,74,75]. Halotolerant Chytridiomycota strains adaptively modulate osmotic pressure and metabolic pathways to improve soil physicochemical properties under high salinity [76]. Future research should focus on functional trait-based consortia and multi-omics approaches to identify key metabolic pathways for targeted strain selection.

## 4. From Insights to Impact: Engineering Sustainable Solutions

**Figure 1:**
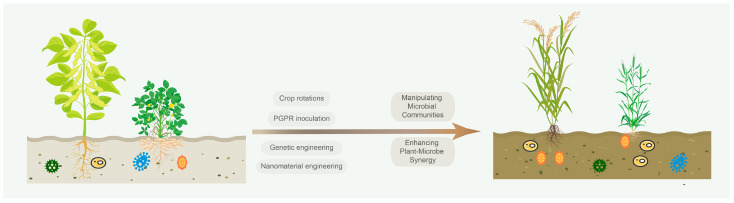
Microbiome engineering strategies based on oilseed crop–microbe interactions for soil restoration.

Traditional approaches for improving crop yield and soil properties historically relied on crop rotation and intercropping. Oilseed crop intercropping systems exhibit remarkable potential for saline–alkali soil remediation by synergistically enhancing soil physicochemical properties and microbial diversity through the adjacent cultivation of diverse species [77,78,79]. For example, the rotation of *Arachis hypogaea* and *Zea mays* optimizes microbial communities, significantly increasing the abundance of beneficial bacteria such as Acidobacteria and fungi such as Ascomycota [80]. Leguminous oilseed crop rotations enhance soil organic carbon, available phosphorus, and total nitrogen by recruiting symbiotic microbes through root exudates [77]. While effective, these conventional methods exhibit limited efficiency, prompting the development of advanced systemic solutions for saline–alkali land restoration (Figure 1).

### 4.1. Microbiome-Based Technologies

PGPR inoculation technology enhances oilseed crop stress resilience by modulating plant–microbe interactions. In saline–alkali environments, PGPR such as Bacillus and Pseudomonas reshape root architecture via indole-3-acetic acid (IAA) secretion, improving water and nutrient uptake [81,82,83]. Biofilm formation and extracellular polymeric substance (EPS) production by PGPR establish protective root layers, enhancing rhizosphere water retention and nutrient availability [7,84,85]. These bacteria also alleviate salt-induced oxidative damage by activating antioxidant gene expression and promoting osmoprotectant synthesis [84,86,87]. Synergistic interactions with arbuscular mycorrhizal fungi such as Glomus further strengthen plant adaptation to saline–alkali stress through enhanced nutrient acquisition and rhizosphere ecological optimization [85,88].

### 4.2. Nanomaterial-Enhanced Remediation

Nanomaterial engineering introduces novel dimensions for microbial–plant collaborative soil remediation. Engineered materials such as nano-silica and carbon nanotubes directly improve rhizosphere conditions by optimizing soil structure through increased water retention and reduced compaction [89,90]. Beyond soil improvement, nanomaterials directly enhance plant development by promoting root growth, increasing photosynthetic efficiency, and improving stress tolerance mechanisms [91]. Their interactions with microbial communities selectively enrich beneficial taxa such as PGPR, suppress pathogens, and enhance nutrient cycling efficiency [92,93,94]. For instance, carbon-based nanomaterials regulate microbial composition to significantly improve nutrient acquisition in oilseed crops [95,96]. Nano-carriers also serve as slow-release fertilizers, sustaining mineral nutrient supply to boost crop yields while improving long-term soil fertility [97]. However, ecological safety assessments through field trials remain critical to clarify nanomaterial environmental behavior and risks [98,99,100].

### 4.3. AI-Driven Precision Management

Artificial intelligence (AI) drives precision in microbial remediation strategies. Machine learning algorithms analyze soil pH, electrical conductivity, and other parameters to predict optimal microbial consortia, such as synergistic Bacillus–Pseudomonas co-inoculation [101,102]. AI-powered optimization algorithms design custom microbial cocktails by evaluating strain compatibility, metabolic complementarity, and halotolerance traits, while clustering algorithms identify previously unknown synergistic relationships between bacteria and mycorrhizal fungi.

Deep learning models integrate multi-omics data such as microbiome profiles, plant phenotypes, and environmental variables to construct digital twin systems for simulating remediation outcomes [102,103]. These predictive models reduce development costs by forecasting microbial combination effectiveness before field testing. Reinforcement learning systems continuously refine inoculant formulations based on performance feedback, creating self-improving remediation protocols. Furthermore, these optimized AI-driven methods contribute to the augmented biosynthesis of long-chain fatty acids in oilseed crops, thereby improving crop yield and quality [104].

Smart sensors enable real-time soil monitoring, dynamically adjusting irrigation and fertilization to maintain optimal microbial activity [103,105]. Future AI-driven solutions will integrate satellite imagery, weather data, and soil sensing networks to deliver site-specific microbial treatments, transcending traditional trial-and-error approaches and enabling intelligent decision-making for large-scale saline–alkali soil management.

## 5. Conclusions

The intricate interplay between oilseed crops and their rhizosphere microbiomes exemplifies nature’s blueprint for sustainable soil restoration in saline–alkali ecosystems. By harnessing the root exudate-mediated recruitment of stress-resilient microbes and leveraging their metabolic versatility—from phosphate solubilization to ROS scavenging—these plant–microbe partnerships redefine soil remediation strategies. The integration of advanced tools such as nanomaterial-enabled nutrient carriers and AI-driven predictive models underscores a paradigm shift from observational ecology to precision microbiome engineering.

However, translating laboratory insights into field efficacy demands rigorous validation. Challenges persist in balancing nanomaterial efficacy with ecological safety, optimizing AI algorithms for heterogeneous soil conditions, and scaling genetically tailored microbial inoculants. Long-term ecological impacts on native soil flora require systematic evaluation, particularly as outcomes may vary significantly under different climatic conditions and soil types. Site-specific optimization of microbial consortia and technologies is essential, as certain soil characteristics may favor specific interventions over others.

Equally important is the comprehensive assessment of biosafety aspects related to products derived from plants grown in remediated soils, ensuring both ecological integrity and regulatory compliance. Future efforts must prioritize the AI-guided discovery of keystone microbial functions, coupled with cross-disciplinary frameworks that unify genetic, environmental, and computational data.

By anchoring innovation in the principles of plant–microbe co-evolution while addressing these ecological and biosafety considerations, this approach not only revitalizes degraded soils but also pioneers a resilient and sustainable agricultural future that harmonizes technological advancement with environmental stewardship.

## Figures and Tables

**Table 1:** Functional traits of rhizosphere microorganisms in major oilseed crops for saline–alkali soil remediation.

Plant Species	Key Microorganisms	Functional Traits	Benefits	Reference
***Brassica napus***	Proteobacteria, Actinobacteria	Organic acid production, metal chelation	P solubilization, toxicity reduction	[49]
***Glycine max***	Bradyrhizobium, Bacillus	N-fixation, biocontrol	Pathogen suppression	[50]
***Arachis hypogaea***	Pseudomonas, Rhizobium	PGPR activity, symbiosis	Growth promotion, N-fixation	[51]
***Helianthus annuus***	Acidobacteria, Saccharibacteria	Osmoregulation, aggregation	Soil structure improvement	[52]

## Data Availability

No new data were created or analyzed in this study.
